# It's never too late to publish an abandoned trial

**DOI:** 10.12688/f1000research.6519.1

**Published:** 2015-05-15

**Authors:** Celia Shiles, Julia Sinclair

**Affiliations:** 1 Southampton General Hospital, Southampton, Hampshire, SO16 6YD, UK; 2 Faculty of Medicine, University of Southampton, Southampton, Hampshire, SO17 1BJ, UK

**Keywords:** Abandoned trials, Science publishing, Meta-analyses

## Abstract

It is estimated that half of all trials have never been published which can lead to patients being denied the most effective treatment and being exposed to unnecessary side effects.  Furthermore the trial participants have been misinformed since the trial results have not contributed to the care of future patients.

However the non-publication of trials is often not due to a deliberate decision to cover up results.  Commonly in academia it is due to more understandable reasons such as researchers having busy clinical posts, moving onto other more demanding projects, changing research areas or starting a family.  This is called the “file drawer” problem.

The examples in this editorial demonstrate that it is possible to go back, even decades later, and make the results available to inform future evidence based medicine.  We call on others to look into their “file drawer” for unpublished trials.

## Article

The process by which a medical intervention is thought to be effective is now well described. Data from trials are collected together after a systematic search of the literature. These are then pooled in a meta-analysis to give an overall indication of efficacy. These results are then collated to inform policy documents, such as guidelines produced by the National Institute for Health and Care Excellence (NICE) in England and Wales. However any meta-analysis is only as good as the trials which it includes. Missing data such as that which comes from unpublished trials is a serious threat to the integrity of any systematic review.

It is estimated that half of all trials have never been published and the incidence of non-publication is roughly similar whether a trial is funded by industry, governments or academia ^1^. Therefore unpublished trials can lead to patients being denied the most effective treatment, or being exposed to ineffective treatments, or to unnecessary side effects. Furthermore the trial participants, who invested their time and placed themselves at potential risk of side effects, being on placebo or an ineffective treatment, have been misinformed since the trial results have not contributed to the care of future patients.

However the non-publication of trials is often not due to a deliberate decision to cover up results. Commonly in academia it is due to more understandable reasons such as researchers having busy clinical posts, moving onto other more demanding projects, changing research areas or starting a family. This is called the “file drawer” problem.

Many steps are currently being taken to address the non-publication of trials, such as enforcing trial registration. However implementing this type of action prospectively will not affect the current evidence base for some time. Therefore reducing publication bias partly depends on researchers pursuing the publication of trials that are sitting in the “file drawer”.

Doshi *et al.* have made a call for action ^2^. They have asked investigators of unpublished trials to either publish their trial within one year or allow their trial data to be made public so that it can be prepared for publication by others. This concept is called ‘Restoring Invisible and Abandoned Trials’ (RIAT). Furthermore the paper sets out a minimum set of criteria for responsible publication of abandoned trials.

The first paper to be published under the RIAT initiative was a trial that was conducted 20 years ago and involved 1447 bowel cancer patients ^3^. Treasure *et al.* contacted the trial investigators to ask for their permission to re-analyse the results and publish the trial. The trial investigators supported this work and now finally the efforts of the trial participants have contributed to the evidence base.

A further example of a recovered trial is a clinical trial on polycythaemia that was not published due to several setbacks including researchers retiring, data being lost during a relocation of its administrative centre, and journals refusing to publish a partially completed trial. Despite all these issues, the results of the trial have now been reported as an attachment to a commentary in the journal *Trials* which is specifically interested in unpublished trials ^4^.

A final example is a randomised controlled trial of Brief Alcohol Interventions ^5^. This trial was not published for 17 years due to researchers moving abroad and taking on other demanding jobs. Therefore, I was asked by the researchers to prepare the trial for publication by updating the introduction and discussion. As a medical student with plenty of time, I was very keen to assist. Now finally the results are available to contribute to future meta-analyses.

These examples demonstrate that it is possible to go back, even decades later, and make the results available to inform future evidence based medicine. We call on others to look into their “file drawer” for unpublished trials. There is plenty of assistance available to make publication as easy as possible, for example by asking others to update the manuscript or publishing through dedicated journals such as *Trials* or *F1000 Research*. It is never too late to publish an abandoned trial.
